# Poor Sleep as a Precursor to Cognitive Decline in Down Syndrome : A Hypothesis

**DOI:** 10.4172/2161-0460.1000124

**Published:** 2013-08-26

**Authors:** Fabian Fernandez, Jamie O Edgin

**Affiliations:** 1 Department of Physiology, Johns Hopkins University School of Medicine, Baltimore, MD 21205, USA; 2 Department of Psychology and Cognitive Science Program, Sonoran University, Center for Excellence in Developmental Disabilities, University of Arizona, Tucson, AZ 85721, USA

**Keywords:** Down syndrome, Alzheimer’s disease, Sleep disruption, Circadian arrhythmia

## Abstract

We propose that sleep disruption is a lever arm that influences how cognition emerges in development and then declines in response to Alzheimer disease in people with Down syndrome. Addressing sleep disruptions might be an overlooked way to improve cognitive outcomes in this population. This article is a contribution to a Special Issue on Down Syndrome curated by the editors of the Journal of Alzheimer’s Disease & Parkinsonism.

Trisomy for human chromosome 21 (Hsa21; Down syndrome, DS) results in a unique trajectory of developmental outcomes for the brain and craniofacial skeleton. In those with DS, the brain’s overall complexity is reduced owing to fewer cells and narrowing within the sulci and gyri that form its outer contour [1, 2]. Local and interregional connectivity may be altered due to weaker insulation of long-range projections by myelin [3–6], and to differences in how synapses and dendrites mature in response to behavioral experience [7, 8]. Infants with DS exhibit smaller neurocraniums at birth and altogether different morphometric relationships between individual bones of the craniofacial skeleton [9]. These divergent craniofacial features impact breathing [10]. Specifically, tissue crowding produced by midface hypoplasia and realigned soft tissue (i.e., constriction of the pharynx and palate, posterior displacement of the tongue, enlarged tonsilsadenoids) curtails airflow through the upper respiratory tract [11–17]. Propensity toward obesity and reduced muscle tone put further strains on the upper airways [18]. As a result of these medical issues, virtually all people with DS will present symptoms associated with obstructive sleep apnea syndrome (OSAS) and sleep fragmentation [18–24]. The loss of sleep quality wrought by OSAS is predicted to contribute to at least some of the everyday intellectual difficulties experienced by people with DS, presenting a case study in how developmental pathways affected by an extra copy of Hsa21 interact at a macro-level to influence cognitive outcomes later in life.

In this review, we discuss recent evidence linking sleep disruption to Alzheimer disease (AD) and cognitive decline in the typical non-DS population, and stipulate that DS is a population in which these elements might converge. Given the high prevalence of OSAS and AD in DS, we propose that sleep disruption could predispose individuals with DS to earlier onset or faster deterioration with dementia. These factors may be inextricably connected to the DS phenotype-to the extent that all future studies on cognitive development from infancy or cognitive decline in DS must take problematic sleep into account.

## Intellectual Disability and Sleep Disturbance: Doubling Down

The neurocognitive profile of DS is characterized, in part, by deficits in the structure-function of an episodic memory network that unifies the frontal and medial temporal lobes [25–33]. Pathology weighs most heavily at the metaphoric poles of this system: the prefrontal cortex (PFC) and hippocampus [34–39]. The frontal lobes of children with DS show truncated growth within the first 6 months of life [1, 2], fewer convolutions in adulthood [36], and shrinkage within the PFC and surrounding tissue during aging [40]. Concomitant with these volume reductions are failures in subcomponents of working memory, including the phonological loop, central executive, and episodic buffer [34, 41–43]. Several neuroimaging and psychometric studies have confirmed that the hippocampus never fully develops in children with DS [44–46] and then atrophies with aging [47–49]. The selectivity with which the hippocampus is disproportionately affected by trisomy 21 is striking given the otherwise global consequences expected of chromosomal triplication. This emphasis suggests that the hippocampus and the circuitry that binds it within the episodic memory network are sites where multiple atypical developmental processes ferment to steer impaired intellectual function in DS. Below we suggest that sleep disturbance and overexpression of the amyloid precursor protein (*APP*) gene on Hsa21 are two factors that further erode the integrity of this memory system.

In the non-DS population, OSAS has been shown to impair performance in neuropsychological assessments of learning and memory. Children or adolescents meeting polysomnographic (PSG) criteria for OSAS score significantly lower on full scale tests of IQ and particular measures of executive function [50–52], while demonstrating reduced metabolic markers in the hippocampus and frontal cortex [52]. Young or middle-aged adults with OSAS also show compromised working memory and attention, and fairly specific deactivation or atrophy in the same brain regions [53–62]. While these data raise the possibility that OSAS contributes to intellectual disabilities in people with DS, research on the relationship between sleep fragmentation and cognitive outcomes in this population is scarce.

Evidence for OSAS (e.g., elevated apnea-hypopnea index, oxygen desaturation) appears as early as infancy in DS, with the majority of toddlers demonstrating sleep fragmentation and arousals by age 2–4 years [18–23]. Longitudinal EEG analyses from birth indicate that infants with DS spend less time in slow wave sleep (SWS) than non-DS infants and exhibit less spindle activity [63]. Miano et al. found that a group of children with DS experienced more fragmented sleep and reduced stage 2 non-rapid eye movement (nREM) sleep relative to a group with Fragile X syndrome (FXS) and controls [64]. Both FXS and DS displayed increased Stage 1 and decreased REM sleep [64]. More recent studies have confirmed the pattern of excessive sleep fragmentation in DS in comparison to individuals with Williams syndrome and typical controls [65]. These data are consistent with those from DS mouse models (e.g., Ts65dn), which show increased wake at the expense of non-REM periods [66]. Declines in SWS have not been consistently reported in DS, however, with one study in adults suggesting greater SWS than typical adult control subjects presenting with OSAS [67]. In order for links to be made between altered neural function in these stages to cognitive outcomes, more work is needed to track the developmental progression of sleep architecture in DS.

Ongoing studies by Edgin et al. [26] have found that deficiencies in SWS are prevalent during childhood and adolescence in those with DS comorbid for OSAS, and that OSAS might explain some of the variability associated with verbal IQ, memory, and executive function in an unreferred DS community cohort (submitted). In this cohort verbal IQ related to SWS. Two other investigations have related sleep disruption to cognitive outcomes in adults with DS, revealing correlations between OSAS and frontally mediated neuropsychological tasks, such as that measured on a nonverbal IQ test (e.g., Raven’s Progressive Matrices) and other assessments of executive function [68, 69].

How does OSAS weaken cognition in DS and non-DS populations? One possibility is that OSAS limits SWS-dependent memory consolidation, a phenomenon where information from recent episodic memories is integrated into preexisting knowledge [70, 71]. The unique brain oscillations witnessed during SWS are thought to provide windows of plasticity that allow for efficient hippocampal-cortical dialogue. When concurrent with reactivation of nascent experiences, they support the strengthening and integration of memory representations within neocortex [70, 71]. In one tangible example of this process, Gais et al. [72] demonstrated that sleep after word-pair learning in typical adults strengthens functional connectivity between the hippocampus and medial PFC 48-h after encoding, but correlates with preferential PFC activation at recall 6 months later [72]. Boosting SWS oscillations artificially with transcranial stimulation enhances retention of paired-associates learning [73]. Similar findings have been documented in children, suggesting that sleep provides a special state that facilitates memory consolidation across development [74–76]. This body of work highlights the importance of SWS and REM periods for consolidating and abstracting information in children and adults. The mechanisms by which poor sleep impairs cognition in DS plausibly reside here.

OSAS might also impact cognition by inhibiting restorative sleep or perturbing homeostatic synaptic scaling, a phenomenon where synaptic strength built-up over the day is downregulated in the evening [77]. Energy substrates like ATP surge during SWS and provide the energetics necessary for immune and nervous system maintenance, and somatic growth and repair [78]. Suppression of SWS interferes with growth hormone secretion and decreases the body’s ability to store glucose effectively, which can indirectly affect learning and memory [79, 80]. Loss of restorative sleep is likely to influence cognitive outcomes in those with DS who are already at risk for a number of metabolic disorders, including obesity and hypothyroidism [81–84]. But this influence is hard to quantify and is not mutually exclusive from many of the other factors discussed in the current review. An OSAS mechanism that has received decidedly more clinical attention for its role in precipitating cognitive decline is brain injury via recurrent intermittent hypoxemia [85]. Hypoxia has been invoked to explain how OSAS accelerates the progression of Alzheimer neuropathology, which first localizes to the frontal cortex and medial temporal lobe, and quickens the transition from mild cognitive impairment (MCI) to dementia recently documented in several large cohorts of typical adults [86–88]. While sleep-disordered breathing and hypoxemia presage the risk of cognitive impairment in older community-dwelling women as measured by the Mini-Mental State Exam [88], a dementia scale, other studies in middle-aged adults suggest that sleep fragmentation (e.g., arousal index, sleep duration) might be just as significant a variable in OSAS related memory problems [62]. Whether sleep fragmentation or hypoxemia accelerates MCI-AD in DS as it does in the general population remains an important unexplored question.

Experiments using *in vitro* assays and mouse models of AD show that hypoxia promotes AD by driving production of Aβ. Hypoxic neurons upregulate the enzymatic activities of β- and γ-secretases, leading to higher APP cleavage, increased Aβ secretion, and self-aggregation of Aβ into amyloid fibrils and thicker plaque deposits (i.e., the classic amyloid hypothesis of AD) [reviewed in 85]. This cascade from hypoxemia → Aβ generation is likely at work in people with DS, and aside from direct hypoxic injury, provides some added functional insight into how OSAS could further debilitate cognition. An evolving understanding of the interactions between sleep and Aβ metabolism in the non-DS brain suggests that the interplay between *APP* gene dosage and OSAS in DS might set in motion cycles of progressively worse developmental and cognitive outcomes.

## Intellectual Disability, Sleep Fragmentation, and Aβ: Spiraling Down

In an elegant series of investigations that have stretched from “bench-to-bedside,” Kang et al. [89] have shown that interstitial fluid levels of Aβ in the hippocampus of wildtype mice and cerebrospinal fluid (CSF) levels in healthy human volunteers oscillate with the circadian or “sleep-wake” cycle [89, 90]. Aβ secretion is highest during the active period and lowest during rest, scaling proportionately to the total time spent awake [89]. Negative correlations between Aβ and time spent asleep are most pronounced during SWS [89]. The association of Aβ with wakefulness stems from the fact that Aβ production in the brain normally occurs in an activity-dependent fashion [91–94]. Local synaptic activity or regional energy use during the active period triggers APP internalization and processing of Aβ for exocytotic release [91–93]. Not surprisingly, parts of the brain exhibiting the most functional connectivity or those that are repetitively tapped like the default mode network tend to demonstrate the highest amyloid plaque burdens upon AD onset [94, 95].

Acute and chronic sleep deprivation studies performed by the Holtzman laboratory in human *APP* transgenic (Tg2576) or double transgenic (APPswe/PS1δE9) mice reveal that prolonged wakefulness over 24 h significantly enhances Aβ levels in the hippocampus, and over 3 weeks, greater plaque deposition in the MTL [89, 90]. Via a destructive positive feedback loop, aggregation of Aβ for longer stretches will then disrupt the sleep-wake cycle, increase time spent awake, and decrease SWS [90]. People with mutations in the presenilin (*PS*) AD susceptibility gene and confirmed Aβ deposition display circadian arrhythmia, and provide some preliminary data hinting that the feedback loop highlighted in mice is also active in humans [90]. The prospect that sleep fragmentation and Aβ may feed off one another in the non-DS population to prime AD is sobering considering that individuals with DS are genetically predisposed to secrete more Aβ and to present with OSAS from the moment they are born.

It is widely understood that those with DS are trisomic for the *APP* gene and overproduce Aβ in quantities that accelerate the formation of plaques in the brain relative to the non-DS population [96]. The developmental timing of these events is sometimes less appreciated. Immunoreactivity for soluble Aβ_1–42_ has routinely appeared in the youngest children with DS evaluated, from 5 months gestation to 3–4 years of age in the temporal cortex [97, 98], to 8 years of age in the parahippocampal gyrus and other areas of the MTL [99]. Enlarged endosomes, a morphological signature of AD connected to Aβ generation in neural cells, are also noted as early as 7 months gestation [100]. Inevitably, diffuse and senile plaque deposits materialize during adolescence and the early twenties in the hippocampus and PFC [101–103].

What becomes *prima facie* obvious is that people affected by DS are possibly AD-presymptomatic their entire lives. The developmental trajectories in the brain that guide expansion of cognitive skill sets and language, already set astray by Hsa21 trisomy, overlap with trajectories that have been more historically associated with mild cognitive impairment. The overlap is conspicuous in slow developing systems like the episodic memory network, which might not even be fully mature by the time “late-life” degenerating processes start that erode tissue integrity and the quality with which information is acquired and consolidated. Because of the biological complexity of DS, changes in other organs such as the skeleton unexpectedly influence these dynamics. Compression of the mid-face triggers OSAS, hypoxemia, and sleep fragmentation during childhood, which might fuel the production of Aβ from more ample sources of APP. The escalating loss of sleep quality that ensues might help to explain the precipitous drop in intellectual function that occurs in adults with DS once AD goes symptomatic. Although theoretical at the moment, this interplay suggests that sleep problems might weigh on cognitive outcomes more significantly in people with DS relative to those with other forms of intellectual disability such as Fragile X or Williams syndrome.

## Conclusion: Down But Not Out

Several interdependent factors undermine the typical establishment of cognitive skills in children with DS and cause their eventual decline into AD. Poor sleep is one of those factors. Here, we provide a hypothesis regarding the extent to which sleep disruption might alter cognitive development and contribute to pathological aging in DS. We point out several potential mechanisms that may relate to neurocognitive dysfunction across the lifespan, including the effects of sleep fragmentation and hypoxemia on Aβ deposition. While sleep disruptions have been widely reported in DS, only recently has OSAS been specifically linked to learning and memory variability. Given the neural penalties that likely accrue from OSAS and sleep fragmentation in DS, more research is needed to determine exactly how and when sleep contributes to cognitive outcomes. OSAS is treatable with surgery to remove the tonsil-adenoids and nCPAP (nasal continuous positive airway pressure). These treatments vary in efficacy and are sometimes difficult to implement. The advent of medical devices that can adapt nCPAP to neonates and young children with DS might be an important advance in addressing sleep-related cognitive outcomes. Garnering hope for treatment promotion, some centers have reported higher levels of adherence to nCPAP [104]. Research programs examining the effects of nCPAP and other medical interventions are now needed at all ages, but especially in younger children, when sleep disruptions might affect developmental trajectories in the brain.

Our main goal in this review is to stir the field to consider sleep when considering life-long cognitive decline in DS. The evidence presented here creates a cause for concern regarding the negative consequences of poor sleep, including the possibility that feedback loops only serve to exacerbate the influence of these factors. In light of the fact that OSAS and sleep fragmentation could possibly result in neural consequences in DS different than those currently targeted by pharmacological and behavioral treatments, the role of poor sleep, and approaches to treating it, merit further attention.
